# Deciphering the single-cell molecular landscape of ampullary cancer: A rare gastrointestinal malignancy

**DOI:** 10.1016/j.isci.2026.116015

**Published:** 2026-05-22

**Authors:** Karina Cancino-Maldonado, Ramiro Fernández, Clémentine Decamps, Jenny Bonifacio-Mundaca, Eloy Ruiz, Sandro Casavilca-Zambrano, Pascal Pineau, Frédéric Lopez, Stéphane Bertani, Juan Pablo Cerapio

**Affiliations:** 1 Centre de Recherches en Cancérologie de Toulouse, Toulouse, France; 2 UMR 152 PHARMADEV, Université de Toulouse, IRD, Toulouse, France; 3 International Joint Laboratory of Molecular Anthropological Oncology, INEN, IRD, Lima, Peru; 4 Instituto Nacional de Enfermedades Neoplásicas, Lima, Peru; 5 Institut Pasteur, Université Paris Cité, Unité « Virus & Stress Cellulaire », Paris, France

**Keywords:** immunology, pathology, precision medicine, bioinformatics, systems biology, cancer systems biology, transcriptomics

## Abstract

Ampullary carcinoma (AC) is a rare epithelial cancer, representing around 0.2% of all gastrointestinal malignancies worldwide, with an estimated incidence of 0.73 per 100,000 people. The rarity of AC, combined with the intricate anatomy of its site of origin, often complicates histopathological diagnosis. Recent advances in molecular research, however, have begun to illuminate its underlying biological complexity. In this study, we present the first comprehensive single-cell transcriptomic atlas of AC, encompassing both malignant epithelial populations and the tumor immune microenvironment (TIME). We identified upregulation of oncogenic drivers previously linked to AC, along with an AC-specific transcriptional program featuring potential biomarkers for minimally invasive diagnostics. Furthermore, we characterized an immunosuppressive TIME, enriched in anti-inflammatory-like tumor-associated macrophages (TAMs) and T cell subsets with limited antitumor activity. Collectively, these findings provide a foundational resource for the identification and validation of candidate biomarkers for early detection and targeted therapies in AC.

## Highlights

• Ampullary carcinoma (AC) diagnosis is challenged by its complex anatomical origin
• We profiled ampullary carcinoma using single-cell transcriptomics and spatial proteomics
• We identified AC-specific transcriptional programs and candidate biomarkers
• AC exhibits an immunosuppressive microenvironment with therapeutic implications

## Graphical abstract

**Figure:**
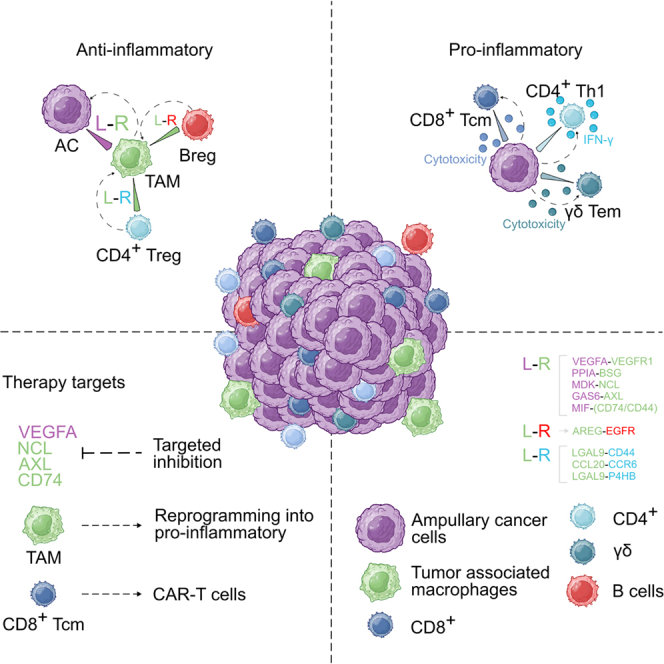

## Introduction

Ampullary carcinoma (AC) is an infrequent epithelial malignancy, accounting for approximately 0.2% of all gastrointestinal (GI) cancers globally.^1^ In countries such as France,^2^ Japan,^3^ Peru,^4^ and the United States,^5^ its incidence is particularly low, around 0.73 cases per 100,000 individuals. Consequently, AC remains understudied, and its epidemiological characteristics and pathogenic mechanisms are poorly understood. Although the literature is sparse, an average annual incidence increase of 0.74% has been observed over the past decade.^2^^,^^3^^,^^5^^,^^6^ AC arises from the ampulla of Vater (AV), a small anatomical structure measuring 2–10 mm in diameter, where the bile and pancreatic ducts converge before emptying into the major duodenal papilla. AV exhibits marked histological heterogeneity, comprising biliary, pancreatic, and intestinal (INT) components.^7^ Owing to this composite tissue origin, AC is frequently misdiagnosed as another periampullary malignancy, such as pancreatic ductal adenocarcinoma (PDAC) or duodenal adenocarcinoma.^7^

Given its rarity and structural complexity, the histopathological classification of AC remains challenging. In 2010, the World Health Organization (WHO) categorized AC into three principal subtypes—pancreatobiliary (PB), INT, and mixed (MIX)—based on epithelial differentiation and immunohistochemical (IHC) profiles.^8^ In 2016, a five-class schema was later proposed to capture a broader spectrum of histological heterogeneity,^9^ although the WHO classification remains the prevailing standard in clinical settings.

To date, histomorphological assessment combined with IHC constitutes the diagnostic gold standard for AC classification. Nonetheless, over the past two decades, recent molecular investigations, although limited in number and scope, have begun to unravel the biological complexity of AC. Most studies have interrogated somatic mutations in canonical oncogenes and tumor suppressor genes—including *KRAS*, *TP53*, *CTNNB1*, and *ELF3*—through analyses of DNA derived from formalin-fixed, paraffin-embedded (FFPE) specimens.^10^^,^^11^ Complementary transcriptomic studies on FFPE and snap-frozen tissue samples have identified upregulation of genes such as *SPP1*, *CD44*, *COL11A*, and *HNF4A*, providing preliminary insights into AC-specific expression patterns.^12^^,^^13^^,^^14^ These efforts have aimed to discriminate AC from other periampullary tumors, propose molecular taxonomies, and uncover prognostic biomarkers.^12^^,^^13^^,^^15^

Within this nascent molecular framework, each histological subtype appears to harbor distinct genomic alterations.^3^^,^^5^^,^^16^ The INT subtype, comprising approximately 56% of cases, demonstrates similarities to colorectal adenocarcinoma, including INT-type glandular architecture, mucinous differentiation, and recurrent alterations in *KRAS*, *TP53*, and *PIK3CA*, alongside microsatellite instability (MSI) features generally associated with a more indolent clinical course. Conversely, the PB subtype resembles pancreaticobiliary carcinomas, exhibits high-grade dysplasia, enrichment for *KRAS*, *TP53*, *CDKN2A*, and *SMAD4* mutations, and an overall less favorable prognosis.^1^^,^^3^^,^^5^

To address the paucity of high-resolution molecular data on AC,^1^^,^^3^^,^^5^ the present study provides the first comprehensive single-cell transcriptomic characterization of malignant epithelial cells and the tumor immune microenvironment (TIME) in AC-INT. Leveraging these data, we further examine the immune checkpoint landscape of this subtype and explore its potential clinical responsiveness to immunotherapies, supported by histopathological validation.

## Results

### Single-cell transcriptomic profiling reveals major epithelial and immune compartments in AC and non-cancer ampullary tissue

**Figure 1:**
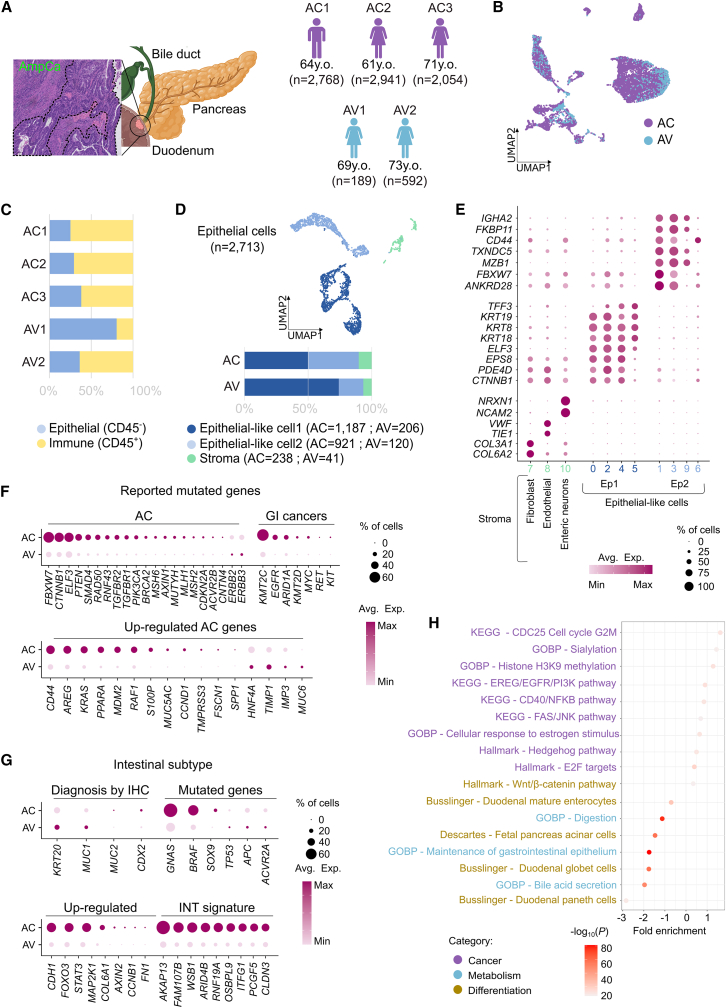
Transcriptomic profile of AC intestinal-like cancer (A) On the left, the tissue location and the tissue structure of AC (HE); and on the right, the cohort information (id, age, and number of cells). (B) UMAP plot of all patients and healthy donors integrated, colored by sample type. (C) Bar plot of immune and epithelial cell proportions by sample. (D) UMAP plot of all epithelial cells showing their main classes (top), and a bar plot of cell proportions for each class by each tissue type independently (bottom). Bubble plots showing the average gene expression of (E) top-expressed genes for each epithelial class and subclass, (F) previously reported AC-associated genes for AC and AV separately, and (G) previously identified genes associated with the AC-INT subtype. (H) Enrichment plot of the most relevant pathway associated with AC tumor-like cells. Ep1, epithelial-like 1; Ep2, epithelial-like 2.

The study included three tumor samples of AC patients and two AV from healthy donors (Figure 1A). The mean age of AC and AV donors was 65.3 ± 5.13 and 71 ± 2.8 years, respectively (*p* > 0.05). Diagnosis and histopathological classification were determined based on hematoxylin and eosin (H&E) staining according to WHO criteria, with all AC cases in the cohort classified as INT subtype (Figure S1A). Serum analyses in all three revealed elevated levels of carcinoembryonic antigen (CEA, >2.5 ng/mL) and cancer antigen 19–9 (CA19-9, >30.9 U/mL), markers commonly associated with colorectal and pancreatic malignancies, respectively. Abnormal metabolic profiles were also observed, including increased concentrations of bilirubin (>22 μmol/L), alanine aminotransferase (ALT, >72 U/L), aspartate aminotransferase (AST, >59 U/L), lactate dehydrogenase (LDH, >3 13 U/L), and gamma-glutamyl transferase (GGT, >73 U/L) (Table 1). Given that MSI is strongly associated with INT subtype, we assessed mismatch repair (MMR) proteins (e.g., MSH2, MSH6, and PMS2) by IHC. Nuclear expression of all MMR proteins was intact, suggesting a low probability of MSI in these AC-INT patients.

**Table 1:** Clinical features

	AC (*n* = 3)	Healthy donor (*n* = 2)
Age (years)		
Mean ± SD	65.3 ± 5.13	71 ± 2.8
Range	61–71	69–73
Gender (n)		
Male	1	–
Female	2	2
BMI		
Mean ± SD	25.4 ± 1.6	–
Range	24–27	–
Diagnostic	adenocarcinoma	AV1: epidermoid carcinoma AV2: kidney carcinoma
Type	intestinal	–
Grade	2	–
Total bilirubin		
Mean ± SD	20.5 ± 6.8	–
Range	13.9–27.6	–
Alkalin phosphatase		
Mean ± SD	263.6 ± 135	–
Range	128–398	–
ALT		
Mean ± SD	89.6 ± 54.9	–
Range	39–148	–
AST		
Mean ± SD	47 ± 17.7	–
Range	31–66	–
GGT		
Mean ± SD	197.6 ± 52.9	–
Range	141–246	–
LDH		
Mean ± SD	163 ± 66.7	–
Range	114–239	–
CA19-9		
Mean ± SD	382.7 ± 654.6	–
Range	3.85–1,138.29	–
CEA		
Mean ± SD	6.2 ± 9.3	–
Range	<0.5–16.96	–

BMI, body mass index; ALT, alanine transaminase; AST, aspartate transaminase; GGT, gamma-glutamyl transferase; LDH, lactate dehydrogenase; CA19-9, carbohydrate antigen 19–9; CEA, carcinoembryonic antigen.

Following preprocessing and quality control, single-cell transcriptomic datasets from AC and healthy AV were integrated, yielding a total of 7,749 single-cell profiles (AC = 7,029; AV = 720) (Figure 1A). Non-linear dimensionality reduction using Uniform Manifold Approximation and Projection (UMAP) enabled visualization of cellular heterogeneity across samples (Figure 1B). Based on *PTPRC* (CD45) expression, two major cell populations were identified: CD45^+^ immune cells (*n* = 5,036) and CD45^−^ epithelial cells (*n* = 2,713) (Figure 1C). The identities of these populations were further validated through canonical markers for immune cells (*CD3E*, *CD4*, *CD8*, *CD79A*, and *MS4A1*) and GI epithelial cells (*HNF4A*, *KRT20*, and *MUC1*) (Figure S1B and Table S1). To gain deeper insight, we next analyzed these two compartments separately.

Given the central role of epithelial differentiation in AC classification and the limited molecular resolution achieved to date, we first focused on the epithelial compartment. Epithelial cells were isolated from each sample, integrated, and subjected to UMAP-based dimensionality reduction with unsupervised clustering (Figure S1C). By analyzing the top differentially expressed genes (DEGs) in each cluster, three primary subclasses emerged: (1) epithelial-like 1 (Ep1, *n* = 1,393), (2) epithelial-like 2 (Ep2, *n* = 1,041), and (3) stromal cells (Figure 1D, *n* = 279). As expected, the vast majority of cells in both AC (89.7%) and AV (88.8%) samples fell into the epithelial-like clusters. Ep1 was defined by canonical epithelial markers (*KRT8*, *KRT18*, and *KRT19*), whereas Ep2 displayed stem-like features (*CD44*) and upregulation of regenerative genes (*FBXW7* and *TXNDC5*) (Figures 1E and S1C). The stromal group was further subdivided into three subpopulations: fibroblasts, marked by the expression of collagen genes (*COL3A1*, *COL6A2*), endothelial cells expressing *VWF* and *TIE1*, and enteric neurons, characterized by the expression of adhesion molecules (*NCAM2* and *NRXN1*).

For stringent malignancy classification, we compared epithelial (Ep1 + Ep2) cells from AC against those from AV. A total of 617 DEGs with log_2_FC > 1 were identified (Table S2) and used to derive a single-cell signature score (Figure S1D). Cells were considered malignant if their score exceeded the third quartile (Q3 = 4.334) of AV scores (Figure S1E). Accordingly, AV cells above Q3 (*n* = 83) and AC cells below Q3 (*n* = 260) were excluded from further analyses. After this filtering step, 1,847 AC and 244 AV epithelial cells were retained for downstream assessment, and their tumoral or healthy status were further validated by inferring the copy number variation (CNV) profile for each sample (Figure S1F).

### Epithelial cell transcriptional rewiring shaped by WNT dependence and COMPASS-mediated reprogramming

Although genomic alterations in AC have been reported, their functional consequences remain poorly understood. We therefore examined whether genes previously reported as mutated in AC (*n* = 21) or in other GI cancers (colorectal cancer, CRC and stomach cancer; *n* = 7) are aberrantly expressed in our single-cell dataset (detailed in Table S1). Our analysis revealed an upregulation of *CTNNB1* and *ELF3* (Figures 1E and 1F, top), two genes previously implicated as cancer driver genes of INT subtype.^10^^,^^11^ Previous studies have shown that *CTNNB1* typically harbors activating mutations in AC,^10^^,^^11^ supporting its role as an oncogene. *ELF3*, in contrast, is more often inactivated by truncating mutations^10^^,^^11^; however, its overexpression in our study suggests an alternative, non-mutational mode of deregulation. Notably, both genes converge on WNT signaling,^17^ underscoring its central role in AC carcinogenesis.

Next, to further characterize the INT molecular profile, we curated a panel of 16 AC markers drawn from prior studies (Table S1). Seventy-five percent (12/16) were significantly upregulated in our cohort (Figure 1F, bottom), including stem-cell-associated *CD44*, and progression markers (*AREG*, *KRAS*, *MDM2*, *PPARA*, and *RAF1*) (Table S1).

To create a transcriptomic analogue to established protein-based diagnostics, we examined four canonical diagnostic markers alongside 14 AC-specific genes (detailed in Table S1). Although 72% were upregulated (log_2_FC > 1), only *BRAF*, *CDH1*, *FOXO3*, *GNAS*, and *STAT3* were expressed in over half of the malignant cells. *STAT3* upregulation further implicates WNT/β-catenin in tumor growth and immune evasion,^10^^,^^15^ while *FOXO3* overexpression hints at AKT pathway inactivity (Figure 1G). Finally, by intersecting the 177-gene INT signature, defined by Overman et al.*,*^15^ with our DEGs (AC vs. AV) and retaining genes present in ≥40% of tumor cells, we derived a distilled nine-gene AC-INT transcriptomic signature, comprising four oncogenes (*AKAP13*, *ARID4B*, *CLDN3*, and *RNF19A*), one tumor suppressor (*FAM107B*), an immune modulator (*ITFG1*), an epigenetic regulator (*PCGF5*), and two novel candidates (*OSBPL9* and *WSB1*) (Figure 1G, bottom).

We then performed gene-set enrichment of single-cell DEGs using MSigDB’s C5, C8, H, and Kyoto Encyclopedia of Genes and Genomes (KEGG) MEDICUS collections to reveal the signaling pathways or biological annotations involved in AC carcinogenesis. AC-INT cells exhibited hallmarks of stemness and proliferation (upregulation of Hedgehog signaling and E2F targets), WNT/β-catenin–driven differentiation, and downregulation of essential genes for normal INT and pancreatic metabolism (Figure 1H).

Immune-evasion axes were highlighted by sialyltransferase upregulation (*ST6GAL1* and *ST3GAL1*),^18^ and pro-tumor pathways, CDC25 cell-cycle activation, EREG/EGFR/PI3K, FAS/JNK, and estrogen signaling via ESR1/ESR2 were also enriched^19^^,^^20^^,^^21^ (Figure 1H). Collectively, these analyses reveal a coordinated network in AC-INT that promotes cellular plasticity (*BCAS3*, *CDKN1A*, *CTNNB1*, *EGFR*, *PIK3CA/B*, *ST3GAL1*, and *VEGFA*), immune escape (*MDM2*, *RELB*, *SERPINB9*, and *TRAF3*), and increases the risk of therapeutic resistance development (*ATR*, *EHMT1*, *ETS2*, and *SETDB2*) (Figure S2).

Crucially, we discovered a coordinated upregulation of *KMT2C/MLL3*, *KDM6A/UTX*, and *NCOA6/ASC2*—three key subunits of the complex of proteins associated with SET1 (COMPASS)-like complex, a Trithorax epigenetic activator that catalyzes H3K4 monomethylation at enhancer regions.^22^ This complex physiologically maintains bile-acid homeostasis in the bile duct^23^ and is often implicated in the development of several malignancies, particularly pancreatic cancer.^24^ While most studies suggest a tumor-suppressive function, our findings point to a distinct oncogenic role in AC-INT. This is driven by gain of function alterations resulting in the overexpression of *KMT2C*, *KDM6A*, and *NCOA6*. This ambivalent behavior of chromatin modifiers in cancer has been previously documented—for instance, in the case of the Polycomb repressive complex 2 (PRC2)—and is thought to reflect their function in maintaining, rather than specifying, the transcriptional state of cancer cells.^25^ Importantly, the aberrant activation of COMPASS-like components highlights *KDM6A* as a promising therapeutic vulnerability in AC.^24^

Nevertheless, in contrast with other neighboring GI cancers such as PDAC (GEO: GSE291124) and CRC (GEO: GSE294559), the AC-INT population displayed both unique features and partially shared transcriptomic commonalities. PDAC shared several transcriptional traits with AC-INT, including upregulation of *AKAP13*, *ARID4B*, *FOXO3*, *ITFG1*, and all markers associated with the COMPASS-like complex (Figure S2D). Concordantly, pathway-level analyses revealed that PDAC samples exhibited enrichment of multiple biological annotations previously implicated in AC carcinogenesis, including pro-tumorigenic signaling pathways, WNT/β-catenin-driven differentiation programs, loss of genes required for normal INT and pancreatic metabolism, and enrichment of immune-evasion-related processes such as sialylation (Figure S2E). By contrast, comparison with CRC revealed no enrichment of the main transcriptomic features defining AC-INT, either at the gene or pathway level (Figures S2D and S2E; see also supplemental information for details). Together, these findings indicate that while AC-INT transcriptomic programs are largely distinct from CRC, they partially overlap with those observed in PDAC, suggesting that anatomically related GI tumors may, in certain extends, converge on shared carcinogenic programs.

### Characterization of adaptive immunity in AC

**Figure 2:**
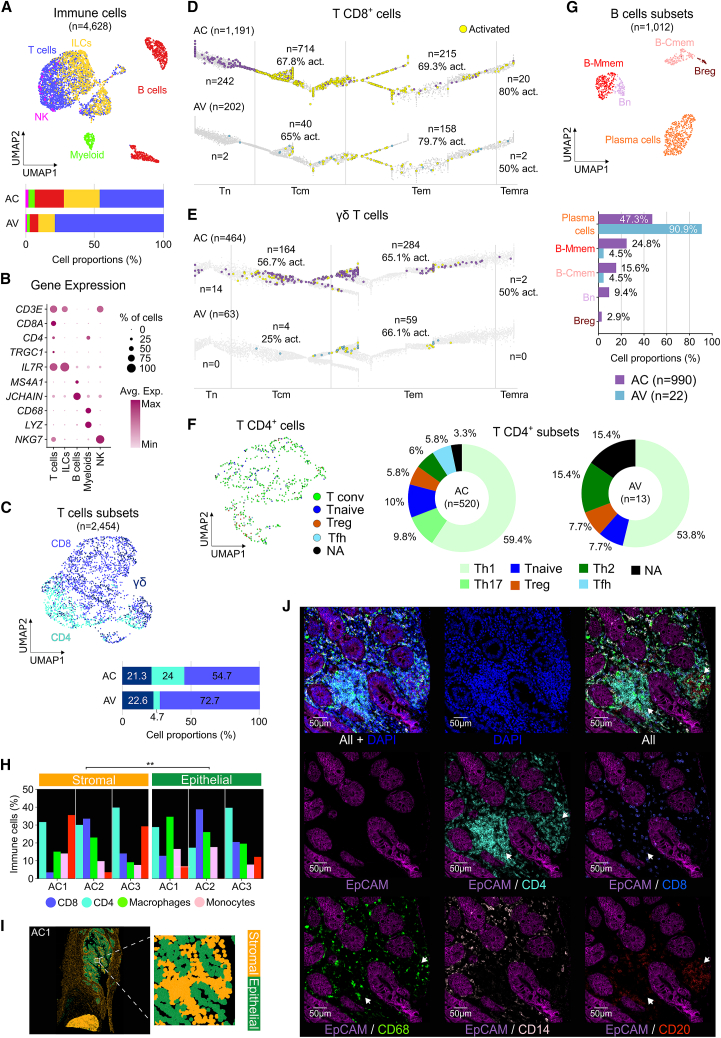
Adaptive immunity landscape of a rare cancer (A) UMAP plot of all the identified immune cells for all patients (AC) and healthy donors (AV) integrated (top), and a bar plot showing the proportion of the main immune cell populations for both AC and AV independently (bottom). (B) Bubble plot showing the average gene expression of canonical immune markers for each main immune cell population. (C) UMAP plot of all identified T cells colored by T cell subsets for AC and AV integrated (top), and a bar plot showing the proportion of all T cell subsets for both AC and AV independently (bottom). Trajectory maps of (D) T CD8^+^ and (E) γδ T cells overlaid on their specific reference differentiation trajectory for AC and AV independently. Color key: non-activated AC cells (purple); non-activated AV cells (light blue); activated cells (yellow). (F) UMAP plot of all T CD4^+^ cells colored by T CD4^+^ subsets for AC and AV integrated (left), and pie charts showing the proportion of T CD4^+^ subsets for AC and AV separately (right). (G) UMAP plot of all identified B cells colored by their subsets for AC and AV integrated (top), and a bar plot showing the proportion of these classes for AC and AV (bottom). (H) Bar plot of immune cell types proportions for both compartments (stromal and epithelial) for each mIF-sample separately. (I) Image plot showing the distribution of both stromal and epithelial compartments on the entire AC1 sample (left) and a zoom in of a region of interest (right). (J) mIF image showing epithelial cells (EpCAM+), T cells (CD8^+^ and CD4^+^), B cells (CD20^+^), and myeloid cells (CD14^+^ and CD68^+^), as well as DAPI as a positive control of the same region of interest in (I). Tconv, conventional T cells; Tfh, follicular helper T cells; Th, helper T cells; Treg, regulatory T cells, ∗∗ chi-squared test *p* < 0.01 (*p* = 0.005).

Using a progressive signature-based strategy (further details in STAR Methods), we classified 5,040 immune cells (AC = 4,687 and AV = 353) into T cells, B cells, natural killer (NK), innate lymphoid cells (ILC), and myeloid lineages (Figures 2A and 2B). T cells were the predominant population infiltrating both tissues (AC = 46.4%; AV = 78.8%), with a significant shift in proportions between tumor and normal samples (*p* = 0.00013) (Figure 2A).

The tumor-specific immune response is led by T cells and B cells. CD8^+^ cells are the subtype of T cells that will specifically recognize tumor antigens, via major histocompatibility complex (MHC) I, and they will kill them via three key cytotoxic immune pathways: granzyme-perforin, Fas/FasL, and IFN-γ.^26^^,^^27^^,^^28^ Accordingly, CD8^+^ cells were the T cell subtype with higher proportions in tumors (AC = 46.4%), but they are also the most abundant in the AV samples (78.8%) (Figure 2C). In addition, using trajectory inference, we identified the differentiation status of these cells (detailed in STAR methods). Most of CD8^+^ T cells in the AC tissue are central memory (Tcm; *n* = 714), in contrast to AV tissue, where most of the CD8^+^ T cells are effector memory (Tem; *n* = 158) (Figure 2D). As it has been previously demonstrated, T cell differentiation states are often associated with specific functional capabilities. For example, Tcm are long-term memory cells that will quickly expand and differentiate into effectors upon tumor reencounter, while Tem cells are able to infiltrate the tumors and mediate an active cytotoxicity.^29^^,^^30^ Nevertheless, if some Tcm are transitioning to Tem, they also express activation markers; therefore, we determined their activation state using two gene signatures (Figures S3A and S3B, Table S3). Interestingly, the proportion of activated cells in the tumor among Tcm and Tem was ∼70%, while in the normal tissue was 65% in Tcm and 80% in Tem. These significantly lower proportions (*p* = 0.03) of activated Tem in the AC tissue maybe associated with the tumor’s immune evasion mechanisms. Although the overall activation proportions are slightly lower in the tumor, the presence of Tcm could be an important feature for immunotherapy durability (e.g., chimeric antigen receptor, CAR T persistence).^31^

Another relevant subtype of T cell that will target tumoral cells is γδ cells. Unlike CD8^+^ T cells, γδ T cells will not recognize peptides via MHC, they will rather recognize stress ligands, phosphoantigens, or lipids.^32^ However, as T CD8^+^, γδ T cells also differentiated into naive (Tn), Tcm, Tem, and terminally differentiated effector memory CD45Ra^+^ (Temra) cells.^33^ Therefore, as mentioned previously, we also used trajectory inference to identify their differentiation status. Both tumoral (AC) and healthy donor (AV) tissues showed ∼20% of γδ T cell infiltration (Figure 2C), and more of them were Tem cells (AC = 284 and AV = 59, Figure 2E). Furthermore, we also investigated their activation state and found that in both cases, ∼60% of γδ Tem cells show activation signals. In addition, most of the activated γδ Tem cells in both tissues are tissue-resident TCRVγnon9 (∼45% on average of activated Tem). No clear difference has been found between AC and AV tissues; however, the high proportion of γδ Tem cells in the tumor may imply an antitumoral role because of their cytotoxicity activity or IFN-γ production. Furthermore, γδ T cells might also be important for developing and monitoring γδ-targeted cancer immunotherapy.^34^

The higher proportion of T CD4^+^ cells in the tumoral tissue (24% vs. 4.7%) showed a more relevant implication of this T subset, in contrast to γδ T, in the AC microenvironment (Figure 2F, left). It has been well-described that the main role of T CD4^+^ is to support CD8^+^ T cells and B cells’ activity.^35^ Depending on the subtype of T CD4^+^ cells, specific anti-tumor (helper T cells [Th] 1 and T follicular helper [Tfh])^35^ or pro-tumor (T regulatory [Treg])^36^ response is associated; however, some subtypes may play dual roles (Th2 and Th17).^37^^,^^38^ We found that most of the CD4^+^ T cells in the tumor were Th1 (59.4%), this type of subset is characterized to be pro-inflammatory (IFN-γ producers), supporting CD8^+^ T cells activity (Figure 2F, right). However, the concomitant presence of Treg, Th2, and Th17 CD4^+^ T cells in sizable proportions might also suggest a pro-tumor signaling, indicating a complex TIME interplay.

Finally, as mentioned before, B cells are also part of the adaptive immunity, which targets tumoral cells. Here, we found a higher proportion of B cells in AC tissue (21.1%) in contrast to AV (6.2%) (Figure 2A); accordingly, it has already been reported that B cells produce tumor-specific antibodies, regulate T cell activation, and support tertiary lymphoid structures (TLS) in the tumors.^39^^,^^40^ Depending on their subtype, activation state, and the tumor microenvironment (TME), they can also promote tumor progression.^41^ As expected, most of the B cells found in the healthy donor tissue were plasma cells (91%), as they play a crucial role in immune homeostasis (Figure 2G). However, in the AC tissue, we found a loss in proportion of plasma cells (47.3%) and an increase in memory (Bmem = 40.4%), naive (Bn = 2.9%), and regulatory B cells (Breg = 2.9%). The increased proportions of Bmem and Bn in the tumoral tissue might be associated with an active immune response against the tumoral cells, as they were barely found in the healthy donor tissue (Bmem = 1 and Bn = 0). Furthermore, a larger proportion (24.8%) of Bmem in the tumor is marginal zone-like (B-Mmem), a subset that rapidly responds to T-independent antigens. In contrast, B class-switched (B-Cmem, 15.6%) require T-dependent antigens and germinal center origin.^42^ The presence of naive B cells, as well as CD4^+^ Tfh (5.8%), could be associated with TLS in the tumor, which is usually associated with better survival in multiple cancers (e.g., lung, melanoma, and breast).^39^ In contrast, the presence of Breg in the tumor has been correlated with poor prognosis and immunotherapy resistance.^41^

To validate our findings, we performed multiplex immunofluorescence (mIF; PhenoCycler Fusion) using nine antibodies on FFPE blocks from the same patient set (see STAR Methods). We delineated epithelial (intra-tumoral) and stromal compartments and annotated major immune cell populations (Figure S3C). Quantitative analysis revealed significant differences in immune cell proportions between compartments (*p* = 0.002). Consistent with single-cell RNA sequencing (scRNA-seq) data, T cells were the predominant immune population across all samples and compartments, followed by myeloid and B cells (Figure S3D). Only one sample (AC2) showed a higher proportion of CD8^+^ over CD4^+^ T cells. Across patients, CD8^+^ T cells were mainly localized within the epithelial compartment, whereas CD4^+^ T cells were enriched in the stroma (Figures 2H–2J), likely explaining the higher CD8^+^ proportion observed in our scRNA-seq dataset, which was primarily derived from intra-tumoral regions. As previously suggested, the presence of B cells and CD4^+^ T cells were associated with TLS formation. In agreement, samples with higher stromal B cell proportions (AC1 and AC3; Figure 2H) also showed increased intra-tumoral B cell infiltration and a greater number of TLS (Figures 2J and S3C).

In summary, our data reveal a complex and dual role of adaptive immune cells within the TME. On one hand, CD8^+^ T cells, γδ T cells, and CD4^+^ Th1 cells exhibit pro-inflammatory features (e.g., IFN-γ), together with the presence of Bmem and TLS, showing an active anti-tumor immune response. On the other hand, CD4^+^ Tregs and Th2/Th17 subsets together with Bregs appear to support a pro-tumorigenic microenvironment, indicating an intricate immune dynamic. Moreover, the detection of active cytotoxic CD8^+^ and γδ T cells within the tumor tissue highlights the potential for responsiveness to immunotherapeutic strategies. One of the major obstacles to successful immunotherapy is T cell exhaustion, frequently mediated by the tumor expression of immune checkpoint ligands such as PD-L1. However, this may be less relevant in AC, as immunohistochemistry analyses showed that 60% of patients with the INT subtype (*n* = 10) were PD-L1 negative (Figure S3E).

### Characterization of innate immunity in AC

**Figure 3:**
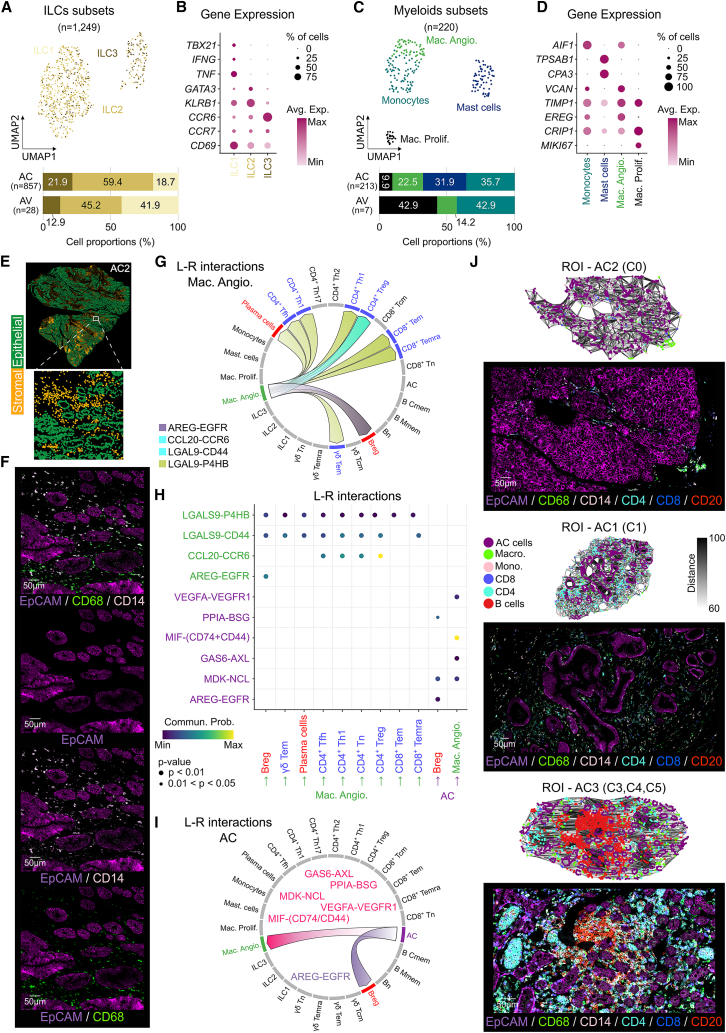
Innate immunity landscape and immune suppressive environment led by anti-inflammatory macrophages (A) UMAP plot of all identified ILCs colored by their subsets for AC and AV integrated (top), and a bar plot showing ILC’s proportions for AC and AV (bottom). (B) Bubble plot showing the average gene expression of the most relevant ILC subsets markers. (C) UMAP plot of all identified myeloid cells colored by their subsets for AC and AV integrated (top), and a bar plot showing the proportion of all myeloid subsets for both AC and AV (bottom). (D) Bubble plot showing the average gene expression of the most relevant myeloid subsets markers. (E) Image plot showing the distribution of both stromal and epithelial compartments on the entire AC2 sample (top) and a zoom in of a region of interest (bottom). (F) mIF image showing epithelial cells (EpCAM+) and myeloid cells (CD14^+^ and CD68^+^) of the same region of interest in (E). (G) Chord diagram of the most relevant ligand-receptor predicted interactions on AC tissue between Mac_Angio and all other subsets, and (I) AC cells and immune subsets. Arrows showed the sense of signaling, and colors are associated with different L-R pairs. (H) Dot plot showing the significant communication probability of all interactions shown in (G and I) for Mac_Angio and AC cells. Arrows showed the sense of signaling. (J) Region of interest (ROI) showing networking between AC cells and immune cells, and a mIF image showing epithelial cells (EpCAM+), T cells (CD8^+^ and CD4^+^), B cells (CD20^+^), and myeloid cells (CD14^+^ and CD68^+^) of the same ROI for a pro-tumoral microenvironment (top), an immune-active region (middle), and a TLS-associated region (bottom). ILCs, innate lymphocyte cells; L-R, ligand-receptor; Avg. Exp., average expression; Commun. Prob., communication probability.

As part of the immune microenvironment, there is also a group of immune cells that do not have a specific anti-tumoral immune response, such as myeloid and ILC. We found a higher proportion of ILCs in the tumor (21.1%) in contrast to the healthy donor’s tissue (11.9%) (Figure 2A). ILCs can also be sub-classified into three subtypes based on their functionality. IL-C1 is mainly associated with an antitumor response (IFN-γ), while IL-C2 is associated with tissue repair, and IL-C3 with maintenance of microbiota homeostasis.^43^ Using gene signatures, we were able to identify these three subtypes (Figures 3A and 3B) and discovered that most of the ILCs in AC were IL-C2 (59.4%), while in the AV tissue, both IL-C1 and IL-C2 were around 43%. It has been reported that the expression of IL-13 and IL-5 by IL-C2 is associated with pro- and anti-tumoral responses, respectively^44^; however, none of samples studied groups expressed either IL-13 or IL-5. Of note, 364 ILCs (41%) have a score of zero for all three gene signatures, so we were incapable to classify them.

In contrast to ILCs, the myeloid compartment was 5-fold lower in the tumor (4.6%), and even lower in the AV tissue (2%) (Figure 2A). To further understand their role in the TME, we identified myeloid subpopulations using previously reported gene signatures (Table S3). Two different subtypes of myeloid cells (monocytes-like and macrophages) in both tissues (AC and AV) were found, and only a one-third (mast cells) in AC (Figures 3C and 3D). However, due to the low number of myeloid cells in AV (*n* = 7), they were excluded from further downstream analyses. Almost equivalent proportions of macrophages (32.4%) and monocyte-like cells (35.7%) were found in AC (Figure 3C). In agreement, we also observed an intra-tumoral high proportion of both macrophages (27%) and monocyte-like cells (14%) using mIF (Figures 2H, 2J, 3E, and 3F). As expected, most of the macrophages found in the tumoral tissue were angiogenic (22.5% of all myeloid). These macrophages are responsible for promoting proliferation and vessel formation via pro-angiogenic factors (e.g., *VEGFA*, *MMP9*, and *TNF*) secretion.^45^ To further confirm their implication on the TME, we used two gene signatures for pro- and anti-inflammatory macrophage profiles (Table S3). We found, in the tumor, that 40% of angiogenic macrophages (Mac_Angio) and 54% of monocyte-like cells share an anti-inflammatory profile (Figure S4A), which could be induced by tumoral cells to reduce the immune response. In contrast, proliferative macrophages (Mac_Prolif) showed a 100% pro-inflammatory profile. Furthermore, mast cells were only found in AC tissue and were highly represented (31.9%) among all the other myeloid subtypes. Interestingly, mast cells also release pro-angiogenic factors, promote immunosuppression (*IL-10*, *TGFB1*, and *PDL1*), and can stimulate tumor cells’ proliferation (c-kit pathway).^46^

In summary, innate immunity seems to play a relevant pro-tumoral role orchestrated by Mac_Angio and mast cells, by promoting tumor proliferation (e.g., *VEGFA*, *MMP9*, and *TNF*) and immunosuppression (e.g., *IL-10*, *TGFB1*, and *PDL1*), respectively. In addition, we believe that ILCs are mainly focused on tissue repair and homeostasis.

### Myeloid-driven signaling networks and immunomodulation in the TME of AC

To further dissect the immune microenvironment, we performed cell-cell communication (CCC) inference using CellChat. Despite their limited abundance, myeloid cells dominated intercellular signaling in AC, while in AV, the CCC was balanced between ILCs, B cells, and T cells (Figure S4B). To delve into the details of these interactions in AC, we performed a refined CCC analysis incorporating all previously defined immune subsets. This analysis revealed that macrophages, among the various myeloid populations, emerged as key communicators, interacting with nearly all immune cell types (Figure S4C).

Our analysis revealed that Mac_Angio cells engage in immunosuppressive crosstalk with CD4^+^ Tregs via C-C motif chemokine ligand (CCL) and GALECTIN pathways, and with Bregs through the epidermal growth factor (EGF) signaling axis (Figure 3G). Both CD4^+^ Tregs and Breg cells have been previously associated with pro-tumoral roles.^36^^,^^41^ More specifically, Mac_Angio engages in signaling with T CD4^+^ Tregs through ligand-receptor pairs CCL20-CCR6 and LGALS9-CD44 (Figures 3G and 3H). Previous studies have shown that tumor-associated macrophages (TAMs) secrete CCL20 and LGALS9,^47^^,^^48^ while CCR6 is found on Tregs, Th17, and B cells, and CD44 is broadly expressed across multiple immune cell types. The CCL20-CCR6 axis is known to promote immune suppression by recruiting Tregs and to support chronic inflammation and angiogenesis by targeting CD4^+^ Th17 cells.^49^ On the other hand, LGALS9-CD44 plays an important role in stabilizing the regulatory phenotype during Treg differentiation.^50^ Furthermore, studies have shown that LGALS9^+^ TAMs suppress anti-tumor immune responses, creating a feedback loop that promotes their pro-tumoral survival.^51^ In addition, within the GALECTIN pathway, we identified a paracrine LGALS9-P4HB interaction involving CD8^+^ (Tem and Temra), CD4^+^ (Tn, Tfh, Th1, and Tregs), γδ T cells (Tem), Bregs, and plasma cells (Figures 3G and 3H). Previous reports in gastric cancer demonstrated that myeloid-derived LGALS9-P4HB interaction enhances cell proliferation, epithelial-mesenchymal transition (EMT), and metabolic reprogramming.^52^ Up to date, there is no evidence of specific ligand-receptor interaction; however, they may support pro-tumoral signals within the TME in AC, as shown in gastric cancer. Finally, within the EGF pathway, we identified a predicted AREG-EGFR interaction between Mac_Angio with Bregs (Figures 3G and 3H). Although a direct link between these two immune subtypes has not been previously reported, EGF signaling in Tregs and cancer associated fibrobalsts (CAF) is known to facilitate immunosuppressive niches.^53^ As in Tregs, AREG^+^TAMs may promote Bregs induction and enhance immune suppression. Finally, as previously noted, mast cells may contribute to pro-tumor signaling, and we identified a potential interaction with CD4^+^ Tregs through the CXCL16-CXCR6 axis (Figure S4D). Even though mast cells typically act as sentinel cells, allowing an effective immune response, within the TME, they can adopt pro-tumoral roles, notably by recruiting immunosuppressive cells such as Tregs.^54^ Accordingly, mast cells can produce CXCL16, either in a membrane-bound or soluble form (sCXCL16), while Tregs express its receptor, CXCR6.^55^ The chemoattractant capabilities of sCXCL16 have been previously demonstrated in glioblastoma.^56^

The polarization of pro-tumoral macrophages, which actively contribute to shaping the TME of AC, is primarily driven by bidirectional signaling with tumoral cells. Accordingly, CCC analysis revealed that AC cells predominantly engage in paracrine communication with anti-inflammatory immune cells, particularly Mac_Angio, through different signaling pathways (e.g., EGF, MK, MIF, CypA, and VEGF) (Figure 3I). Moreover, the specific receptor-ligand pairs involved in these pathways have all been previously implicated in driving macrophage polarization toward an immunosuppressive phenotype and in facilitating an immunosuppressive TME. For instance, we again identified AREG-EGFR interaction (Figures 3H and 3I), which, as mentioned previously, initiates signaling pathways that facilitate the establishment of immunosuppressive niches by targeting Breg.^53^ While AREG expression by tumor cells has been previously demonstrated,^57^ no study to date has reported direct interactions between tumor cells and immune cells mediated via this signaling axis. In addition, we also found other ligand-receptor specific interactions between AC cells and Mac_Angio, such as GAS6-AXL, MDK-NCL, MIF- (CD74^+^CD44), PPIA-BSG, and VEGFA-VEGFR1 (Figures 3H and 3I). All these ligand-receptor pairs also target the activation of macrophage immunosuppressive polarization,^58^^,^^59^^,^^60^ by triggering different complementary mechanisms. While AXL and CD74^+^CD44 trigger PI3K/AKT-NF-κB, MEK/ERK signaling pathways,^58^^,^^60^ NCL, BSG, and VEGFR1 mediate downstream immunosuppressive signaling via TGF-β.^59^^,^^61^^,^^62^ However, they also trigger specific pathways, such as increased IL-6 production by NCL,^63^ and IL-10 production by both BSG and VEGFR1.^61^^,^^62^

Finally, we analyzed the gene expression of all the ligand-receptors pairs examined previously (Figure S4E). Consistent with our previous observations, all Mac_Angio cells showed high expression of *CD74* and *CD44*, while 50% strongly expressed *CCL20*, and only 25% expressed *AXL* and *LGALS9* at high levels. In contrast, 50% of tumor cells exhibited high expression of *MDK* and *VEGFA*, whereas only 25% expressed *GAS6*. These AC cells also expressed *MIF* (75%), *PPIA* (50%), and *AREG* (50%), though at lower levels compared to the aforementioned genes. Additionally, CD4^+^ Tregs and Breg cells displayed high expression of *CCR6* (75%) and *EGFR* (25%), respectively.

Moreover, neighborhood enrichment analysis combined with network clustering of the mIF data revealed the coexistence of immune exclusion niches and structured TLS (Figures 3, S4F, and S4G), confirming a heterogeneous immune landscape in which anti-tumoral immune activation (i.e., high TIL density and TLS presence) and spatial immune restriction (i.e., low TIL density and absence of TLS) occur together (see also supplemental information for details). Consistent with our previous observations, immune-restricted regions were characterized by an increased abundance of TAMs, suggestive of a pro-tumoral microenvironment (Figure 3J, top). In contrast, immune-active regions (Figure 3J, middle) were predominantly enriched in T cells and monocytes, supporting a cytotoxic and potentially anti-tumoral immune response. TLS-associated regions (Figure 3J, bottom) exhibited the highest proportions of tumor-infiltrating lymphocytes (TIL), with a marked enrichment of B cells (CD20^+^) and CD4^+^ T cells as defining features.

In summary, here we depicted multiple signaling pathways collectively contributing to an immunosuppressive microenvironment, primarily driven by macrophages and tumoral cells crosstalk. Furthermore, these ligand-receptor interactions represent potential therapeutic targets in AC. Notably, several of these ligand/receptors (AXL, NCL, CD74, and VEGFA) have already been proven to reverse their immune suppressive effect upon targeted inhibition in other cancer types.^62^^,^^63^^,^^64^^,^^65^^,^^66^ Nevertheless, the organized immune spatial structures within the TME also supports the biological relevance of these findings, and highlights confirm the potential opportunities for therapeutic targeting of immune interactions.

## Discussion

Few studies have explored the transcriptomic landscape of AC. While some have underscored its molecular distinction from other cancers,^13^ others have focused on intra-tumoral heterogeneity across subtypes.^12^^,^^14^^,^^15^ However, a definitive molecular classification remains unresolved.^15^ To better characterize this malignancy, we included AV tissue from healthy donors as a baseline. We confirmed the upregulation of key oncogenic drivers previously associated with AC, including *CTNNB1*, *ELF3*, *FOXO3*, and *STAT3*. Furthermore, we report the first AC-specific transcriptomic signature, encompassing newly identified oncogenes (*AKAP13*, *ARID4B*, *KAT6A*, and *RNF19A*), a tumor suppressor (*FAM107B*), an immune modulator (*ITFG1*), and an epigenetic regulator (*PCGF5*). Together with known deregulated genes, these candidates may serve as potential arkers for less invasive diagnostic approaches.

Our findings also implicate coordinated dysregulation of the WNT/β-catenin, PI3K/AKT, Hedgehog, and the COMPASS-like complex in AC development and progression. The WNT/β-catenin axis is a well-established oncogenic driver,^67^ and therapeutic targeting is actively under investigation.^68^ In addition, the COMPASS complex has also been implicated in the development of other cancer types,^24^ and one of its components (*KDM6A*) has emerged as a promising therapeutic target in pancreatic cancer.^24^

In parallel, we characterized a profoundly immunosuppressive TME dominated by anti-inflammatory-like TAMs, which are known to promote tumor growth, angiogenesis, and immune evasion.^69^^,^^70^ Their central role has spurred therapeutic strategies aimed at either depleting these cells or reprogramming them into a pro-inflammatory phenotype.^71^ Although some subsets of T cells (CD8^+^ T cells, γδ T cells, and CD4^+^ Th1) suggests an active anti-tumor immune response, their limited activation in an immunosuppressive TME reduces their chances of actively controlling tumor progression. These findings suggest that therapies enhancing CD8^+^ T cell cytotoxicity (e.g., cytokine therapy, adoptive cell transfer, or CAR T) could be effective,^31^^,^^72^ especially in the context of PD-L1-negative tumors. Thus, dual immunotherapeutic strategies targeting both anti-inflammatory-like TAMs and CD8^+^ T cells may offer enhanced efficacy. Furthermore, the γδ-targeted cancer immunotherapies might also be a key point to explore.^34^

Finally, we acknowledge that future work should aim to include additional AC subtypes and larger cohorts. Nonetheless, our findings provide foundational insights into the molecular and immune landscape of this malignancy and may inform the development of novel therapeutic strategies.

### Limitations of the study

The most relevant limitation of this study is the limited number of samples and low cell yield, which is mainly due to the rarity and heterogeneity of AC. In addition, the small anatomical size of the AV also limited the number of recovered cells.

## Resource availability

### Lead contact

Requests for further information and resources should be directed to and will be fulfilled by the corresponding author, Juan Pablo Cerapio (juan-pablo.cerapio-arroyo@inserm.fr).

### Materials availability

This study did not generate new unique reagents.

### Data and code availability

• The scRNA-seq data generated in this study have been deposited at Gene Expression Omnibus (GEO), and interactively on UCSC Cell Browser (http://cells.ucsc.edu), and are publicly available as of the date of publication. Accession numbers are listed in the key resources table.
• This study did not generate new code; the used codes are already cited in STAR Methods.
• This study did not generate any other item.

## Acknowledgments

The authors thank all patients and donors whose participation was essential to this study. They acknowledge the staff of the 10.13039/100032227Genomics Core Facility at CRCT and the National Tumor Bank at INEN for providing full access to their technical platforms. They also thank E. Sarot and C. Valle from the transcriptomic platform for their support during the single-cell experiments. The authors also appreciate the technical support on spatial proteomics of Y. Nicaise and L. Ligat. The authors also acknowledge M. Ayyoub and J. Guillermet for suggestions and critical comments on the manuscript, as well as colleagues at CRCT for helpful discussions.

This project was supported by the International Joint Laboratories (LMI) program of the French National Research Institute for Sustainable Development (IRD). It also received funding from ITMO Cancer of the French National Alliance for Life Sciences and Health (Aviesan) and the French National Cancer Institute (10.13039/501100006364INCa), with funds administered by the French National Institute of Health and Medical Research (10.13039/501100001677Inserm) under grant agreement 21CD025-00 (to S.B.). It also was support by ECOS-Nord initiative under grant agreement P25S01 (to J.P.C.). K.C.M. was supported by a doctoral fellowship from the research grants for a thesis in the South (ARTS) program of IRD (A02022), and by the 10.13039/501100001665French National Research Agency (ANR) through the Graduate School of Research in Cancer, Ageing, and Rejuvenation (EUR CARe), as part of the Investments for the Future Initiative (grant agreement ANR-18-EURE-0003).

## Author contributions

R.F., S.B., and J.P.C. conceived the study; J.P.C. and S.B. designed the study; R.F. collected the samples, with input from E.R. and S.C.-Z., K.C.M., and J.B.M. processed the samples, with input from S.B., S.C.-Z., and J.P.C., K.C.M. generated the scRNA-seq data and performed bioinformatic analyses, both with input from J.P.C.; figures were prepared by K.C.M. with input from J.P.C.; data were interpreted by K.C.M. and J.P.C., with input from P.P. and S.B., C.D., E.R., and F.L. contributed to critical discussions; the manuscript was written by K.C.M. and J.P.C., with input from P.P. and S.B. All authors approved the final version.

## Declaration of interests

The authors declare no competing interests.

## STAR★Methods

### Key resources table

**Table:** 

REAGENT or RESOURCE	SOURCE	IDENTIFIER
**Antibodies**		

PD-L1 (Clone 22C3)	Dako	M3653; RRID: AB_2833074
CD3e	Akoya Bioscience	AKYP0062; RRID: AB_3094503
CD4	Akoya Bioscience	AKYP0048; RRID: AB_3665715
CD8	Akoya Bioscience	AKYP0028; RRID: AB_2915960
CD14	Akoya Bioscience	AKYP0079; RRID: AB_3083457
CD20	Akoya Bioscience	AKYP0049; RRID: AB_3094498
CD68	Akoya Bioscience	AKYP0050; RRID: AB_2935894
Pan-Cytokeratin	Akoya Bioscience	AKYP0053; RRID: AB_3662772
EpCAM	Akoya Bioscience	AKYP0119; RRID: AB_2935888

**Biological samples**		

Ampullary cancer	Patients	AC1, AC2, AC3
Ampulla of Vater	Healthy donors	AV1, AV2

**Critical commercial assays**		

Chromium Next GEM Single Cell 3ʹ GEM(Dual Index)	10x Genomics	PN-1000130
Library construction kit	10x Genomics	PN-1000196
Chromium Next GEM Single 3′ Gel Bead Kit v3.1	10x Genomics	PN-1000129
Dynabeads™ MyOne™ SILANE	10x Genomics	PN-1000048
Chromium Next GEM Chip G Single Cell Kit	10x Genomics	PN-1000127
Dual Index Kit TT set A	10x Genomics	PN-1000215
Sample Kit for PhenoCycler-Fusion	Akoya Bioscience	AKYP7000009

**Deposited data**		

GEO	This study	GEO: GSE308520
GEO	Pancreatic ductal adenocarcinoma (PDAC)	GEO: GSE291124
GEO	Colorectal cancer (CRC)	GEO: GSE294559
UCSC Cell Browser	This study	human-ampullary-cancer
Reference human genome	GRCh38-2020	https://www.ncbi.nlm.nih.gov/datasets/genome/GCF_000001405.26/
Multigene signatures	MSigDB	https://www.gsea-msigdb.org/gsea/msigdb/collections.jsp
Immune cell signatures	Cerapio et al.^74^	https://www.tandfonline.com/doi/full/10.1080/2162402X.2021.1939518
Myeloids cell signatures	Guimarães et al.^73^	https://www.nature.com/articles/s41467-024-49916-4
List of DEGs (intestinal subtype vs. pancreatobiliar subtype)	Overman et al.^15^	https://journals.plos.org/plosone/article?id=10.1371/journal.pone.0065144

**Software and algorithms**		

CellChat (v2)	Jin et al.^74^	https://github.com/jinworks/CellChat
Cell Ranger (v7.0.1)	10x Genomics	https://support.10xgenomics.com/single-cell-gene-expression/software/downloads/7.0.1
Dplyr (v1.1.4)	CRAN	https://cran.r-project.org/web/packages/dplyr/index.html
Ggplot2 (v3.5.2)	CRAN	https://cran.r-project.org/web/packages/ggplot2/index.html
GraphPad Prism v10.3	GraphPad	https://www.graphpad.com/features
MuSpAn	Bull et al.^75^	https://www.muspan.co.uk
Python (v3.10.16)	Python™	https://www.python.org
R (v4.4.3)	R project	https://www.r-project.org/
Seurat (v5.3)	Hao et al.^76^ - CRAN	https://cran.r-project.org/web/packages/Seurat/index.html
Single-Cell Signature Scorer tool	Pont et al.^77^	https://sites.google.com/site/fredsoftwares/products/single-cell-signature-explorer
Squidpy	Palla et al.^78^	https://squidpy.readthedocs.io/en/stable/
UCell	Andreatta et al.^78^	https://github.com/carmonalab/UCell?tab=readme-ov-file

### Experimental model and study participant details

This study included three ampullary cancer patients and two healthy donors. The mean age of patients and healthy donors was 65.3 ± 5.13 and 71 ± 2.8 years, respectively. The sex ratio in patients was of 2 females and 1 male, while both healthy donors were female. Non sex bias was observed. Diagnosis and histopathological classification were determined based on hematoxylin and eosin staining according to WHO criteria, with all tumoral samples in the cohort classified as intestinal subtype. Serum analyses showed elevated levels of carcinoembryonic antigen (CEA, >2.5 ng/mL) and cancer antigen 19-9 (CA19-9, >30.9 U/mL). Abnormal metabolic profiles were also observed, including increased concentrations of bilirubin (>22 μmol/L), alanine aminotransferase (ALT, >72 U/L), aspartate aminotransferase (AST, >59 U/L), lactate dehydrogenase (LDH, >3 13 U/L), and gamma-glutamyl transferase (GGT, >73 U/L). Further details in Table 1 and results.

### Method details

#### Ethics statement

This project received clearance from the Institutional Review Board of the National Cancer Institute of Peru (INEN), under approval number 388-2024-CIEI/INEN. All procedures strictly adhere to the ethical principles outlined in the WMA Declaration of Helsinki on medical research involving human participants.

#### Patient recruitment

Patients were excluded from the present cohort if they were diagnosed with neuroendocrine tumors, mixed carcinomas, sarcomas of the ampulla of Vater, or received treatment. All enrolled patients and healthy donors provided informed consent.

#### Immunohistochemistry (IHC)

A tissue microarray (TMA) of formalin-fixed, paraffin-embedded (FFPE) tissues was prepared by the National Biobank of Peru at INEN according to an established procedure. Next, TMA slides were processed following DAKO’s automated immunohistochemistry protocol. First, heat-induced epitope retrieval was performed using the PT Link automated antigen retrieval system. Following this step, slides were washed with buffer before proceeding to automated antibody staining on the Autostainer Link 48 (Dako). Briefly, primary and secondary antibodies were applied, and the antigen–antibody interaction was visualized using the chromogen 3,3′-diaminobenzidine (DAB). Once staining was complete, the slides were prepared for dehydration, clearing, and mounting. Finally, all tissue immunohistochemistry was reviewed at the INEN Department of Pathology.

#### Sample collection

Samples were collected from surgical remnants following a pancreaticoduodenectomy in patients diagnosed with ampullary carcinoma (AC). Ampulla of Vater tissue (AV) samples were collected from healthy donors through GI tract endoscopy. Briefly, patients diagnosed with non-GI cancers and scheduled for ambulatory endoscopy were invited to participate in this study by donating a portion of their healthy ampulla. Pathological diagnosis of AC was confirmed by the Anatomopathology department of INEN, using the recommended classification by the WHO (pancreatobiliar - PB, intestinal – INT and mixed - MIX).

#### Tissue processing

After surgery, samples were taken to the National Tumor Biobank at INEN, where they were enzymatically dissociated using the Tumor Dissociation Kit (Miltenyi Biotec). Briefly, small tissue fragments (2-4 mm) were dissociated in rotation (MACSmix Tube Rotator) using gentleMACS Dissociator and an enzymatic cocktail at 37°C for 60 min. After enzymatic digestion, cell suspension was filtered (70 μm) and washed with RPMI-1640 medium (Sigma-Aldrich). Next, Red Blood Cell Lysis Solution (10X) (Miltenyi Biotec) was used according to the manufacturer’s instructions to eliminate erythrocytes. Finally, cells were recovered after centrifugation. Cell viability and concentration for each cell suspension were automatically assessed using trypan blue staining with the Countess II Automated Cell Counter (Thermo Fisher Scientific). The cells were then gradually cryopreserved at −150°C in RPMI-1640 supplemented with 10% dimethyl sulfoxide (DMSO) (Sigma-Aldrich).

#### Single-cell RNA sequencing

Samples were prepared following the user guide “Chromium Next GEM Single Cell 3′ Reagent Kit v3.1 (Dual Index)” and loaded into the Chromium X (10x Genomics). After thawing, cell suspensions were filtered (40 μM), followed by two sequential washing steps (RPMI-1640 medium +10% FBS), and resuspended in cold PBS 1X. Cell suspensions of approximately 20,000 cells were obtained, and cell viability was manually evaluated using trypan blue staining. Only samples with more than 50% of cell viability were further used for a droplet-based single-cell capture protocol. Briefly, each cell was tagged by a bead containing unique molecular identifiers (UMI) in an oil droplet (Gel Beads in Emulsion, GEM). Then, following reverse transcription, cDNA was extracted from each GEM, and its quantity and quality were assessed. Next, based on the calculated cDNA concentration per sample, appropriate modifications to the PCR cycles of scRNA-seq library preparation were carried out, and each scRNA-seq library’s molarity and quality were determined. Finally, a sequencing depth of 20,000 read pairs per cell was considered for each run.

#### Multiple immunofluorescence (PhenoCycler fusion)

High-plex spatial proteomic imaging was performed on FFPE slides (4 μm) from ampullary carcinoma samples. Slides preparations were used according to the manufacturer’s instructions. Briefly, after the deparaffinization and tissue hydration, the tissue was stained by our 9-antibody panel. For Akoya Biosciences’ antibodies, the dilution factor used was 1:200. Following slide staining, the slides were preserved until fluorescence detection on the PhenoCycler Fusion system. During each detection cycle, fluorescence from three antibody reporters was captured. Additionally, two detection cycles for DAPI staining were included in the detection. Images were acquired automatically at each detection round, and the resulting datasets were compiled into a single high-resolution composite file (.qptiff) for downstream spatial proteomic analysis.

### Quantification and statistical analysis

#### Data pre-processing

After sequencing, bcl2 files were converted to FASTQ format by using the cellranger-mkfastq algorithm (10x Genomics). Then, reads were aligned to the reference genome GRCh38, and the UMI x cell expression matrix for each sample was generated using the Cell Ranger Count pipeline (10x Genomics). Data analysis was performed in R using the Seurat pipeline v5.^76^ Cells with more than 20% mitochondrial DNA (cell death markers) and more than 5,000 features per cell (doublets) were removed. All samples were merged, preprocessed and normalized before the next steps.

#### Cell annotation

Initial cell annotation was based on *PTPRC* (CD45) expression, classifying cells into immune (CD45^+^) and epithelial (CD45^−^) compartments, which were subsequently analyzed independently. Epithelial cells were annotated based on the most significant differentially expressed genes (DEGs) for each cluster. Immune cells were annotated using a previously reported progressive signature-based strategy,^79^ in which myeloid, T, and B cell populations were first identified based on signature scores and a gating strategy. Then, using the same strategy, we further identified the T cell subsets. However, myeloid and B cell sub-populations were identified using previously reported marker genes.^73^^,^^80^

#### Single-cell signatures scoring

To determine the single-cell score for a specific gene-set (or pathway), we used both the Single-Cell Signature Scorer tool^77^ and the R library UCell.^81^ Multigene signatures were either downloaded from MSigDB (https://www.gsea-msigdb.org/gsea/msigdb/collections.jsp) or obtained from previously reported publications,^73^^,^^79^ further details in Table S1. Finally, for single-cell scores visualization and analysis, each cell’s UMAP coordinates and metadata were merged.

#### Single-cell copy number variations (CNV) inference

In order to confirm the tumor origin of the isolated epithelial cells from patients, we determine the CNV architectures at both the single-cell and sample levels using SCEVAN package for each patient (AC) and healthy donor (HD). Briefly, following the default methodology reported for SCEVAN package, we extracted the cells-by-gene count matrices for each sample independently and used the multiSampleComparisonClonalCN function, with HD cells as normal cells, to estimate CNV profiles at both the single-cell and sample levels.

#### Pseudotime trajectories

As described before,^82^ we used the R packages Dynverse, Seurat and single_cell_Injection to determine the differentiation status of CD8^+^ and γδ T cells. Briefly, first we integrate our dataset (Query) into a reference dataset (reference) in which cell coordinates are already annotated (UMAP, trajectory, milestone, and pseudotime). The resulting dataset (Query 2) is then used by Seurat’s function MapQuery to anchor Query 2 in the “reference”. The reference cell matrix of CD8^+^ and γδ T has been previously published.^79^ Thus, the pseudotime and Time clusters for each T cell subset were determined, which will finally allow us to obtain the differentiation status.

#### Cell–cell communication

In order to investigate the cell-cell communication of our different cell subtypes, we used CellChat v2 (R package) to infer cellular interactions.^74^ This tool determines the probability that a group of cells expresses a ligand (average gene expression, “triMean”) and another group of cells its corresponding receptor (min. cells = 10), using a ligand-receptor database (CellChatDB). Then, we determined the strength/significance interaction and associated key signaling networks. Finally, we selected the biologically relevant Ligand-Receptor interactions taking into account the literature and the biological evidence of this interaction.

#### Spatial proteomic imaging analysis

Images were analyzed through sequential steps of segmentation, fluorescence-based cell annotation, and evaluation of patterns (spatial distribution) across the tissue. First, images were imported into QuPath, and fluorescence intensity for each antibody was quality controlled before segmentation in each tissue independently. Nuclei were segmented using DAPI staining and the StarDist algorithm in Qupath.

Following segmentation, the resulting single-cell matrix was exported for cell annotation using FlowJo, and the following main cell types were identified: tumor cells (EPCAM^+^ and PanCytokeratin^+^), T cells (CD3e^+^/CD8^+^ and CD3e^+^/CD4^+^), Myeloid cells (Monocytes: CD14^+^/CD68^+^ and Macrophages: CD14^−^/CD68^+^), and B cells (CD20^+^). Then, the data was prepared for neighborhood network clustering using MuSpAn toolbox.^75^ The networking method used was Proximity with a max edge distance of 100, and for the clustering the used method was minibatch K-means, with 6 clusters. Resulting distance matrix was then used to create a cluster map for complementary understanding. The same data was also evaluated using neighborhood enrichment analysis via Squidpy.^78^ First, we computed a spatial graph with generic coordinate type with eight neighborhoods, then se applied the neighborhood enrichment analysis using 1,000 permutations. Finally, we used the obtained Z-scores to further analyze the positive or negative enrichment between cell types.

#### Statistical analysis

Means differences between groups were assessed by ANOVA or *t* test using Prism v10.3 (GraphPad). Proportions comparisons were assessed by or Chi^2^ also using Prism v10.3 (GraphPad). If it is not precise otherwise in the manuscript, for the statistical analyses, we considered a *p* < 0.05 as significant (∗: *p* < 0.05; ∗∗: *p* < 0.005, ∗∗∗: *p* < 0.0005, ∗∗∗∗: *p* < 0.0001). For the multigene signatures scores the adjusted considered *p*-value was less than 5.46 E^−06^.

#### Data visualization

Data visualization was carried out using the Single-cell Multilayer Viewer,^79^ R, and Prism v10.3 (GraphPad). For Spatial proteomic visualization, we used QuPath, Seurat (R package) and Python (MuSpAn and Suidpy).

## Supplemental information

Document S1. Figures S1–S3

Table S1. List of genes used in this work with their respective public reference

Table S2. 617 Differentially expressed genes (DEGs) between epithelial cells from AC and AV

Table S3. Gene signatures used in the present work
