# Supplementary material for: Deciphering the single-cell molecular landscape of ampullary cancer: A rare gastrointestinal malignancy
Source: iScience. 2026 May 22;29(6):116015. doi: 10.1016/j.isci.2026.116015 (PMC13223973; doi:10.1016/j.isci.2026.116015)
Supplement: Document S1. Figures S1–S3 [file mmc1.pdf]

iScience, Volume *29*

Supplemental information

Deciphering the single-cell molecular landscape of ampullary cancer: A rare gastrointestinal malignancy

Karina Cancino-Maldonado, Ramiro Fernández, Clémentine Decamps, Jenny Bonifacio-Mundaca, Eloy Ruiz, Sandro Casavilca-Zambrano, Pascal Pineau, Frédéric Lopez, Stéphane Bertani, and Juan Pablo Cerapio

Supplemental information

DATA S1

Additional characterization of pancreatic ductal adenocarcinoma (PDAC) and colorectal cancer (CRC) transcriptomes, associated with the section "*Epithelial cell transcriptional rewiring shaped by WNT dependence and COMPASS-mediated reprogramming*"

To assess whether transcriptional programs identified in AC are disease-specific or shared across neighboring gastrointestinal malignancies, we integrated our AC scRNA-seq dataset with publicly available single-nucleus RNA-seq data from pancreatic ductal adenocarcinoma (PDAC, n = 17) and scRNA-seq data from paired colorectal cancer (CRC) and adjacent non-tumoral samples (n = 18).

Among previously reported AC markers significantly upregulated in the AC-INT, only AREG was significantly downregulated (log2FC > 1) in both CRC and PDAC relative to AC-INT (Figure S2d). In addition, among genes previously established as protein-based diagnostic markers for AC, both CRC and PDAC—when independently compared with AC—showed significant downregulation of *GNAS*, whereas *FOXO3* was strongly upregulated exclusively in PDAC (Figure S2d).

Analysis of the AC-INT transcriptional signature showed that none of its component genes were upregulated in CRC relative to matched non-tumoral tissue. In contrast, because PDAC samples lack paired non-tumoral controls, direct comparison with AC identified three significantly upregulated genes in PDAC (*AKAP13*, *ARID4B*, and *ITFG1*) (Figure S2d).

Pathway enrichment analyses revealed limited overlap between CRC and AC. In CRC, only CDC25-mediated cell-cycle activation was enriched relative to adjacent non-tumoral tissue. By contrast, PDAC displayed enrichment of multiple pathways previously associated with AC, including CDC25 cell-cycle activation, EREG/EGFR/PI3K signaling, and FAS/JNK signaling. PDAC samples also showed enrichment of WNT/β-catenin–driven differentiation programs, loss of genes involved in normal intestinal and pancreatic metabolism, and enrichment of immune-evasion–related processes such as sialylation (Figure S2e).

Consistent with one of the main findings of this study, genes associated with the COMPASS-like complex were selectively enriched. All three COMPASS-related genes upregulated in AC relative to ampulla of Vater (AV) tissue were also upregulated in PDAC, whereas no such enrichment was observed in CRC (Figure S2d).

Collectively, these analyses indicate that the transcriptomic features defining the AC-INT are largely specific when compared with CRC, even when analyzed to its respective non-tumoral counterpart. In contrast, PDAC shares several transcriptional programs with AC, suggesting partial convergence of carcinogenic mechanisms across anatomically related gastrointestinal tumors.

Spatial immune microenvironment structure of the ampullary carcinoma, associated to section "*Myeloid-driven signaling networks and immunomodulation in the tumor microenvironment of ampullary carcinoma*"

Neighborhood enrichment analysis (Figure S4f) demonstrated that, within the epithelial compartment, immune cells exhibited pronounced negative enrichment relative to tumor cells (92% on average across patients), reflecting spatial segregation between these populations. This pattern is consistent with an immune-excluded or an immune-desert phenotype. In contrast, such spatial avoidance was not detected

within the stromal compartment. Notably, within the epithelial compartment, immune cell subsets displayed strong positive enrichment with each other, suggesting coordinated clustering. Conversely, in the stromal compartment, we observed preferential co-localization between myeloid and T cells.

To further depict spatial organization across compartments, we conducted neighborhood network clustering integrating epithelial and stromal regions (Figure S4g and Figure 3i). Cluster 0 was predominantly enriched in tumor cells and showed strong negative enrichment of immune cells, reinforcing the presence of immune exclusion niches (Figure 3i, top). On the other hand, Cluster 1 displayed marked positive enrichment among tumor cells, monocytes, and CD8+ T cells, indicating spatial proximity between malignant cells and cytotoxic immune populations within tumor regions (Figure 3i, middle).

Additionally, three clusters (Clusters 3, 4, and 5) were associated with tertiary lymphoid structures (TLS) (Figure 3i, bottom). These clusters revealed layered spatial organization. Cluster 4 was enriched in B cells, $CD8^+$ T cells, and $CD4^+$ T cells and exhibited strong interaction with tumor cells. Cluster 5 was predominantly enriched in B cells and macrophages and was typically positioned adjacent to tumor-interacting regions. Cluster 3 was enriched in $CD4^+$ T cells and macrophages.

SUPPLEMENTARY FIGURES

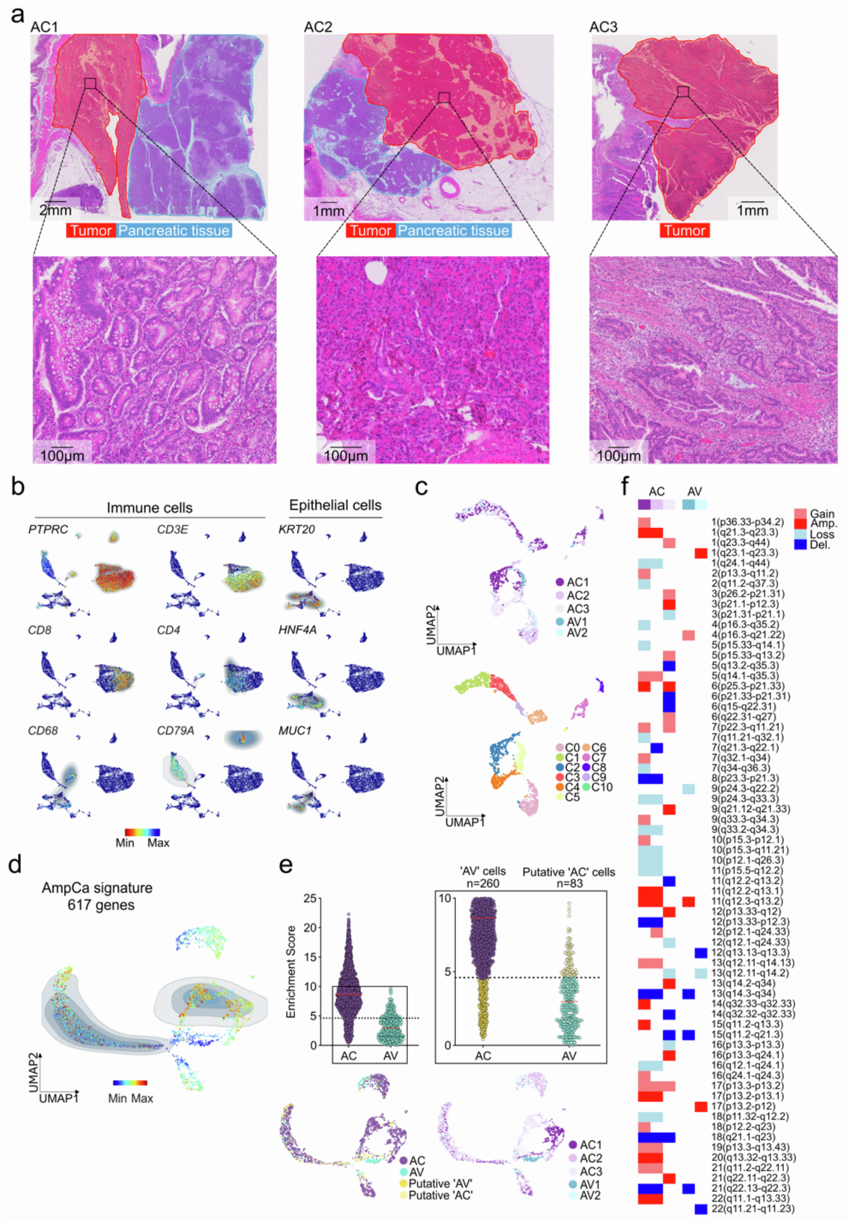

Figures S1. Uncovering malignant cells, related to Figure 1. **(a)** Hematoxylin-eosin (HE) images of the entire section (top), and a representative region of interest (ROI) of each AC sample included in this study. **(b)** UMAP plot showing the gene expression of both canonical immune and epithelial genes of all patients and healthy donors integrated **(c)** UMAP plot of all epithelial and stromal cells colored by sample type (top) and by cluster (bottom). **(d)** New UMAP of only all epithelial cells, without stromal cells, showing the "AmpCa signature" single-cell scores. **(e)** Violin plots showing the stringent malignancy classification based on the "AmpCa signature" by tissue type (top-left). Cells in yellow were those excluded from further analyses (top-right). In addition, UMAP plots of only all epithelial cells colored by real and putative epithelial cells (bottom-left) and by sample type (bottom-right). **(f)** CNV estimation average by sample. The sample colors as in (c). Amp.: Amplification; Del.: Deletion.

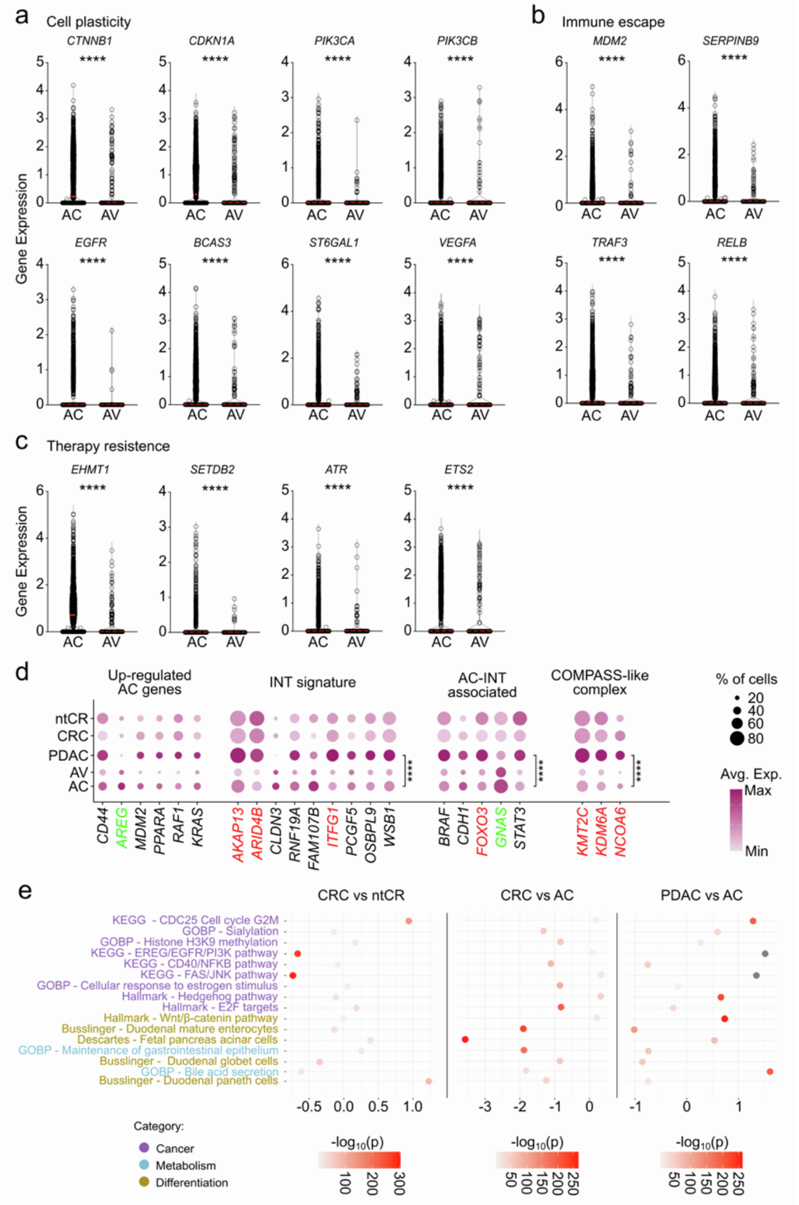

Figures S2. Epithelial cells' transcriptional features, related to Figure 1. Violin plots showing the relative gene expression of genes associated to **(a)** cell plasticity, **(b)** immune escape, and **(c)** to an increase risk of therapeutic resistance. **(d)** Bubble plots showing the average gene expression of previously reported AC-associated genes for AC, AC-INT signature, previously identified genes associated with the AC-INT subtype, and associated genes to COMPASS-like complex (from left to right) for adjacent-non-tumoral colorectal (ntCRC), colorectal cancer (CRC), pancreatic ductal adenocarcinoma (PDAC), ampulla of Vater (AV), and ampullary cancer (AC) single-cell transcriptomic data. Gene names in red: upregulated in PDAC; in green: downregulated in PDAC; significant contrasts are represented by **** (t-test $p < 0.0001$). **(e)** Enrichment plot of the most relevant pathway associated with AC tumor-like cells for three different contrasts: CRC vs ntCR, CRC vs AC and PDAC vs AC. INT: intestinal.

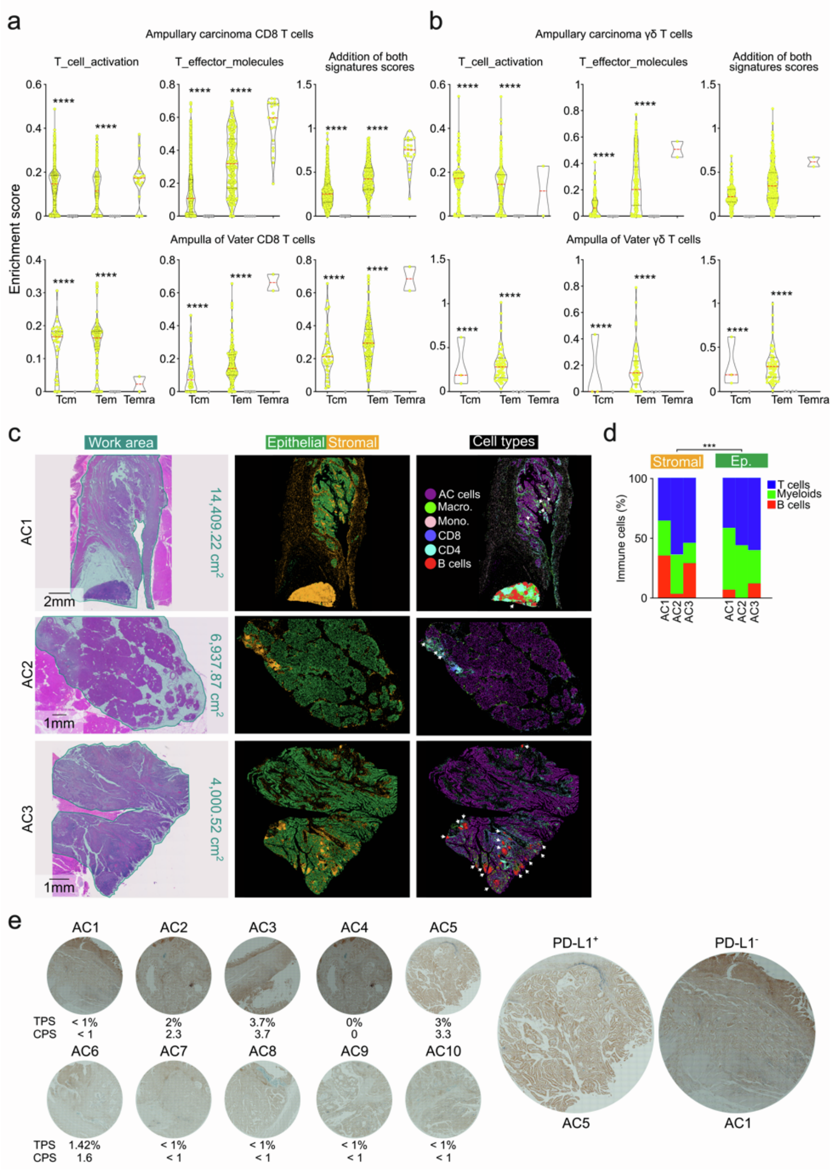

Figures S3. Tumor immune microenvironment, related to Figure 2. Violin plots showing the scores of the gene signatures used to determine the activation state of central-memory, effector-memory, and effector-memory CD45Ra^{+} **(a)** CD8^{+} and **(b)** γδ T cells for ampullary carcinoma (top) and ampulla for Vater (bottom) tissues. ****: t-test $p < 0.0001$**.** **(c)** Hematoxylin-eosin (HE) image showing the selected work area (left), image plot showing the distribution of both stromal and epithelial compartments (middle), and the AC and immune cells distribution in the tissue (right) for each mIF-sample. Arrows (white in the right panel) in this figure indicate the presence of TLS in the tissue. **(d)** Bar plot of the immune cell proportions in both compartments (stromal and epithelial) for each mIF-sample separately**.** **(e)** Immunohistochemistry of PD-L1 Tissue microarray of AC-INT patients, their respective tumor proportion score (TPS) and the combined positive score (CPS) at the bottom of each spot. TPS: < 1% = No PD-L1 expression, 1%-49% = Low PD-L1 expression, >50% = high PD-L1 expression; CPS: < 1 = Negative, ≥ 1 = Positive, ≥ 10 = Positive (high expression).

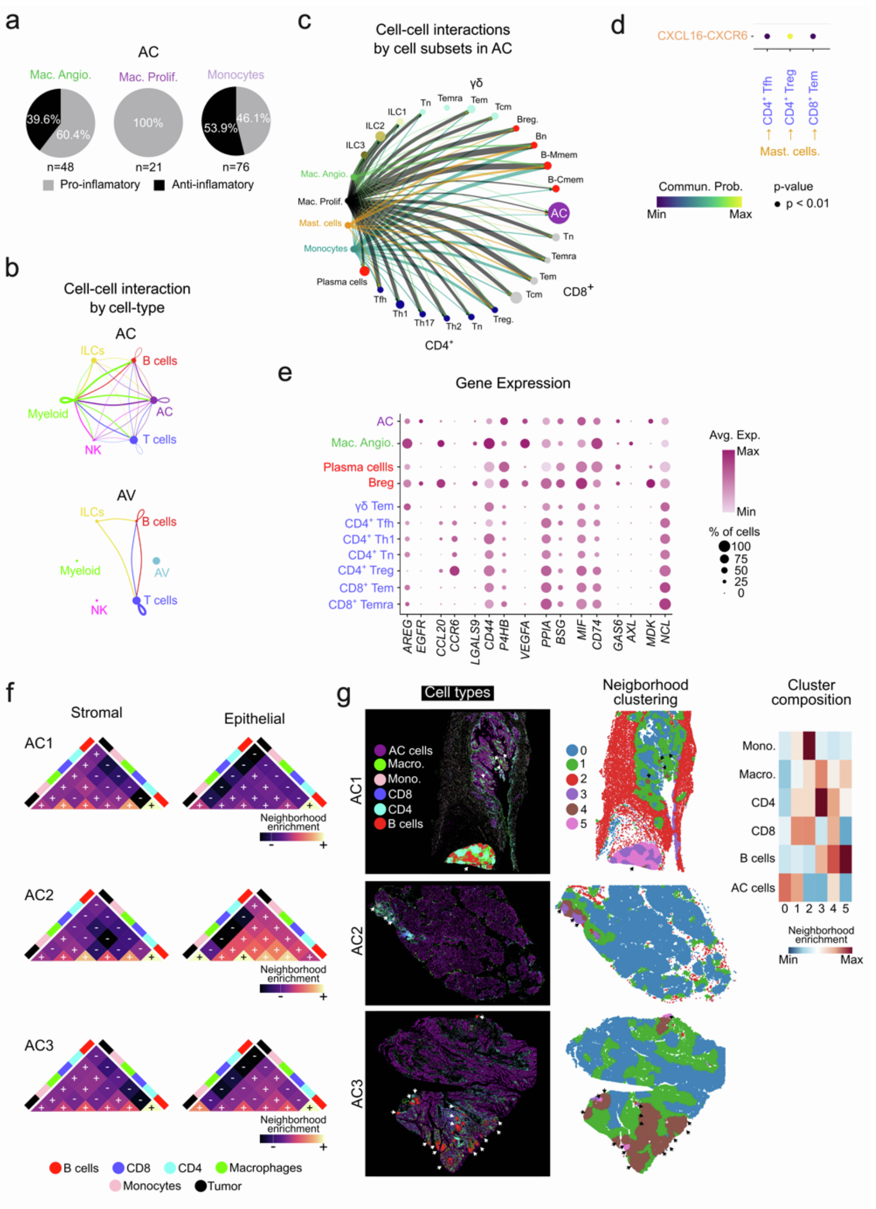

Figures S4. Tumor immune microenvironment networking, related to Figure 3. (a) Pie chart showing the proportions (%) of Pro- and Anti- inflammatory by Myeloid subsets in AC. **(b)** Circle plot showing cell-cell predicted interactions between the main immune cell populations with AC (top) and AV (bottom) cells independently. Line color is associated with each cell type (AC: purple; AV: light blue; B: red; T cells: blue; Myeloid: green; and ILCs: yellow), and line thickness represents the weight or strength of the interaction. **(c)** Circle plot showing cell-cell predicted interactions in AC between all Myeloid subsets and all immune cell sub-populations. **(d)** Dot plot showing the significant communication probability of Mast cell interactions displayed in figure (c)**.** Arrows showed the sense of signaling**.** Mac. Angio = angiogenic macrophages; Mac. Prolif. = proliferative macrophages. **(e)** Bubble plot showing the average gene expression of L-R genes explored in Figure 3h, for each cell subset involved in CCC interaction. ILCs: Innate lymphocyte cells, L-R: Ligand-receptor, Avg. Exp.: Average expression, Commun. Prob.: Communication probability. **(f)** Pyramidal

matrix plot showing the neighborhood enrichment distance between all cell types (AC and immune cells) for each mIF-sample separately. **(g)** Image plot showing AC and immune cells distribution in the tissue (left), and the neighborhood clustering (middle) for each mIF-sample separately. In addition, on the right panel, a matrix plot shows the overall neighborhood enrichment composition of each identified cluster (middle panel) for each cell type (left panel). Arrows (white on the left panel, and black on the middle panel) in these figures indicate the TLS presence in the tissue.
